# Miscellany

**Published:** 1893-05

**Authors:** 


### Miscellany.

### THE PAN-AMERICAN MEDICAL CONGRESS.

OFFICE OF THE SECRETARY-GENERAL, 311 ELM ST.,
CINCINNATI, APRIL 2, 1893.

(*Official Bulletin.*)

*The Executive Committee of the First Pan-American Medical Congress promulgates the following information:*

1. The first Pan-American Medical Congress will be opened under the Presidency of Prof. William Pepper, M. D., LL. D., President of the University of Pennsylvania, at Washington, D. C., September 5th, and will adjourn September 8, 1893.

2. The countries officially participating in the Congress are restricted to Argentine Republic, Bolivia, Brazil, British North America, British West Indies, (including B. Honduras,) Chile, Dominican Republic, Honduras (Sp.), Mexico, Nicaragua, Paraguay, Peru, Salvador, Republic of Columbia, Republic of Costa Rica, Ecuador, Guatemala, Haiti, Kingdom of Hawaii, Spanish West Indies, United States, Uruguay, Venezuela, Danish, Dutch, and French West Indies.

Distinguished representatives of the profession from other countries are expected to be present as guests and to participate in the proceedings.

3. The general sessions will be limited in number, one for opening and one for closing the Congress, being all that will be held unless some necessity arises for a change in this particular. This arrangement will permit members to employ all of the time in the scientific work of the sections, which are as follows :

(1) General Medicine, (2) General Surgery, (3) Military Medicine and Surgery, (4) Obstetrics, (5) Gynecology and Abdominal Surgery, (6) Therapeutics, (7) Anatomy, (8) Physiology, (9) Diseases of Children, (10) Pathology, (11) Ophthalmology, (12) Laryngology and Rhinology, (13) Otology, (14) Dermatology and Syphilography, (15) General Hygiene and Demography, (16) Marine Hygiene and Quarantine, (17) Orthopedic Surgery, (18) Diseases of the Mind and Nervous System, (19) Oral and Dental Surgery, (20) Medical Pedagogics, (21) Medical Jurisprudence, (22) Railway Surgery.

The evenings will be devoted entirely to social features, the detailed announcements of which will be made by the Committee of Arrangements.

4. Membership is limited to the members of medical profession of the Western Hemisphere, including the West Indies and Hawaii, who shall either register at the meeting or shall serve the Congress in the capacity of foreign officers. No membership fee will be accepted from any member residing outside the United States. The membership fee for residents of the United States is ten dollars ($10.00). All registered members will receive a copy of the transactions. Prominent students of the allied sciences will be cordially received as guests and as contributors to the proceedings upon invitation by the Executive Presidents of sections. Ladies' tickets will be issued upon application to registered members only, and will entitle the holders to reduced fare and to admission to all entertainments. *Physicians of the United States should register at once, by remitting $10.00 to Dr. A. M. Owen, Treasurer, Evansville, Indiana.*

5. Papers are solicited, the hope being entertained that the programme will be largely taken up with contributions from outside the United States. Papers may be read in any language, but a copy must be furnished for publication in either Spanish, Portuguese, French, or English, and must not occupy more than twenty minutes in reading. An abstract, not exceeding 600 words, must be furnished the Secretary-General, in one of the above four languages, by not later than July 10th. Abstracts will then be translated by the Literary Bureau into the three remaining languages, and will be published in book-form before the meeting of the Congress.

6. The Congress of the United States has adopted a joint resolution, whereby all the Governments of the Western Hemisphere have been invited by the President to send delegates to the First Pan-American Medical Congress, and has appropriated a liberal sum for the purposes of entertainment.

7. The reduced fare offered by all transportation companies on the occasion of the World's Columbian Exposition, to be held in Chicago, will be open to all persons attending the Pan-American Medical Congress. The Committee of Arrangements will endeavor to secure still greater reduction to members traveling between Chicago and Washington, and an effort will be made to arrange either excursions or circular tours for those who may desire to visit the great universities of the United States. All such arrangements are open to subsequent announcement.

8. By arrangement with the Committee at Rome, the date of the Eleventh International Medical Congress has been so appointed that those who attend the meeting of the Pan-American Medical Congress may subsequently attend the former. The Pan-American Medical Congress will adjourn on the afternoon of September 8th; a steamship will sail from New York on the following day, going by the Azores and Gibraltar, and enabling the tourist to reach Rome on the morning of September 20th, where the Eleventh International Congress will be opened on the afternoon of September 24th. It will thus be seen at a glance that in the period usually allotted to a Summer vacation, the medical tourist may spend a week at the World's Columbian Exposition, the next at the Pan-American Medical Congress, the next week and a half with delightful companions in a voyage to the Mediterranean, the next few days in witnessing the sights of Rome, and in the following week at the Eleventh International Medical Congress. Special reduced rates for members and their families are given both ways on the trip to Rome, particulars of which will be furnished on application to the Secretary-General, 311 Elm street, Cincinnati, Ohio, who is also a member of the American Committee of the Eleventh International Congress.

9. The best possible arrangements will be made with the excellent hotels with which the national capital is abundantly supplied. The Committee of Arrangements will do its utmost to secure desirable rates and locations for members and their families. The headquarters of the Committee of Arrangements is at the Arlington Hotel, where communications may be addressed either to Dr. Samuel S. Adams, Chairman, or Dr. J. R. Wellington, Secretary.

10. Copies of the official announcement of the Congress, containing the regulations and the names of all officers and committeemen of the General Congress and of the various sections, and residing in the various countries, may be obtained upon application to the Secretary-General, or to either of the members of the International Executive Committee, as follows:

Argentine Republic, Dr. Pedro Lagleyze, Calle Artes 46, Buenos Aires; Bolivia, Dr. Emilio di Tomassi, Calle Ayacucho 26, La Paz; British West Indies, Dr. James A. de Wolf, Port of Spain; British North America, Dr. James F. W. Ross, 481 Sherborne St., Toronto; Chili, Dr. Moises Amaral, Facultad de Medicina, Santiago; Costa Rica, Dr. Daniel Nuñez, San José; Dominican Republic, Dr. Julio Leon, Santo Domingo; Ecuador, Dr. Ricardo Cucalon, Guayaquil; Guatemala, Dr. José Monteros, Avenida Sur No. 8, Guatemala City; Haiti, Dr. T. Lamothe, Rue du Centre, Port au Prince; Hawaii, Dr. John A. McGrew, Honolulu; Honduras (Spanish), Dr. Geo. Bernhardt, Tegucigalpa; Mexico, Dr. Uomás Noriega, Hospital de Jesus, Mexico; Nicaragua, Dr. J. I. Urtecho, Calle Real, Granada; Paraguay——; Peru, Dr. Manuel C. Barrios, Facultad de Medicina, Lima; Republic of Colombia, Dr. P. M. Ibañez, Calle 5a Numero 99, Bogota; Salvador, Dr. David J. Guzman, San Salvador; Spanish West Indies, Dr. Juan Santos Fernandez, Calle Reina No. 92, Havana; United States of America, Dr. A. Vander Veer, 28 Eagle street, Albany, N. Y.; United States of Brazil, Dr. Carlos Costa, Rua Largo da Misericordia 7, Rio de Janeiro; Uruguay, Dr. Jacinto de Leon, Calle de Florida No. 65, Montevideo; Venezuela, Dr. Elias Rodriguez, Caracus.

By the Executive Committee,

CHARLES A. L. REED,

*Secretary-General.*

